# A Case of Congenital Nephrotic Syndrome with Crescents Caused by a Novel Compound Heterozygous Pairing of *NPHS1* Genetic Variants

**DOI:** 10.1155/2024/5121375

**Published:** 2024-02-27

**Authors:** Kyle N. Goodman, Pongpratch Puapatanakul, Kevin T. Barton, Mai He, Jeffrey H. Miner, Joseph P. Gaut

**Affiliations:** ^1^Department of Pathology and Immunology, Washington University School of Medicine in St. Louis, St. Louis, MO, USA; ^2^Division of Nephrology, Washington University School of Medicine in St. Louis, St. Louis, MO, USA; ^3^Division of Nephrology, Hypertension and Apheresis, St. Louis Children's Hospital, St. Louis, MO, USA

## Abstract

Congenital nephrotic syndrome is an autosomal recessive inherited disorder that manifests as steroid-resistant massive proteinuria in the first three months of life. Defects in the glomerular filtration mechanism are the primary etiology. We present a child who developed severe nephrotic syndrome at two weeks of age and eventually required a bilateral nephrectomy. Genetic testing revealed compound heterozygous variants in *NPHS1* including a known pathogenic variant and a missense variant of uncertain significance. Light microscopy revealed crescent formation—an atypical finding in congenital nephrotic syndrome caused by nephrin variants—in addition to focal segmental and global glomerulosclerosis. Electron microscopy showed diffuse podocyte foot process effacement. Confocal and Airyscan immunofluorescence microcopy showed aggregation of nephrin in the podocyte cell body that is not a result of diffuse podocyte foot process effacement as seen in minimal change disease. These findings confirm the novel variant as pathogenic.

## 1. Introduction

Congenital nephrotic syndrome (CNS) is characterized by steroid-resistant nephrotic range proteinuria that occurs in the first three months of life. CNS affects approximately 1 to 3 per 100,000 children worldwide [1]. Classically, the disease is caused by homozygous variants in *NPHS1*, the gene located on chromosome 19 that encodes nephrin, a major podocyte structural protein important for forming and maintaining the slit diaphragm. The highest incidence of CNS occurs in Finland. The most frequently described variant in this population is Fin-major, a *NPHS1* frameshift variant leading to a truncated nephrin protein at 90 amino acids and loss of expression by immunofluorescence microscopy in homozygous patients [2, 3]. More than 100 additional variants in *NPHS1* have been reported with variable effects on nephrin structure [4]. Patients with CNS are susceptible to multiple severe complications such as thromboses, failure to thrive, recurrent infection, and hemodynamic instability. Most children develop kidney failure within two to three years [5]. In this report, we describe a case of severe CNS in a patient with compound heterozygous variants in *NPHS1*, including a previously described pathogenic variant [2] inherited from the father and a variant of uncertain significance inherited from the mother. The patient eventually required a bilateral nephrectomy to manage the condition while awaiting kidney transplantation.

## 2. Methods

Illumina Clinical Services Laboratory performed genetic testing for CNS via the TruGenome Undiagnosed Disease Test, which was conducted on the patient and both parents. After the patient underwent a bilateral nephrectomy, the kidneys were fixed in 10% formalin. Representative tissue blocks were further processed, paraffin-embedded, sectioned, and hematoxylin and eosin (H&E), periodic acid-Schiff (PAS), Masson's trichrome, and toluidine blue stained at the Barnes-Jewish Hospital Laboratory and the Anatomic and Molecular Pathology Core Histology Laboratory at the Washington University School of Medicine in St. Louis. Microscopic images of the stained slides were acquired with an Olympus bright-field microscope outfitted with a DP23 digital camera to record images of the tissue sections at 100X to 400X magnification. Electron microscopy was performed on paraffin-embedded tissue at the Electron Microscopy Core Facility at the Washington University School of Medicine in St. Louis, and digital images were obtained with a high-resolution CCD camera. For immunofluorescence microscopy, 5 *μ*m tissue sections were obtained and deparaffinized. Sections were then blocked with 2% bovine serum albumin (BSA) in phosphate buffer saline (PBS). For immunolabeling, sections were incubated with rabbit anti-human nephrin (1 : 300, MAB42693 R&D Bio-Techne) and guinea pig antisynaptopodin-IN (1 : 200, 03-GP94-IN ARP) in 2% BSA overnight at 4°C to outline the slit diaphragm and podocyte foot processes, respectively. Sections were washed with PBS, incubated with appropriate fluorophore-conjugated secondary antibody, washed again with PBS, and mounted with antifade mounting medium (Invitrogen®, P36934). Fluorescence images were acquired with a Zeiss LSM 880 Airyscan two-photon confocal microscope.

## 3. Case Presentation

A 10-day-old male was taken to the emergency room due to seizure-like activity and edema. He was found to have substantial proteinuria, hypoproteinemia, hypoalbuminemia, hypogammaglobulinemia, hypocalcemia, and anemia. CNS was confirmed by 24-hour urine protein analysis. Presentation was complicated by extensive cerebral venous thromboses extending from the posterior superior sagittal sinus into the straight sinus and internal cerebral veins. The patient had a prolonged course in the neonatal intensive care unit. He was anticoagulated with warfarin and started on levetiracetam and phenobarbital, which led to resolution of the thromboses and seizures, respectively. He was anemic, requiring multiple blood transfusions, epoetin alfa therapy, and ferrous iron supplementation. Moreover, nephrotic range proteinuria was managed with daily albumin infusions, furosemide, spironolactone, and intravenous immunoglobulin (for hypogammaglobulinemia). He also had hypothyroidism that was managed with levothyroxine. The patient was discharged almost two months later. Seizure medications were eventually discontinued, but he remained on warfarin for anticoagulation as well as multiple supportive treatments including daily albumin infusions, furosemide, lisinopril, iron and copper supplementation, levothyroxine, and epoetin alfa injections. Due to failure to thrive despite gastrostomy tube placement, failure of medical nephrectomy with persistent massive proteinuria, and complications of peritoneal dialysis (pleuroperitoneal leak), the patient underwent a bilateral nephrectomy at two years of age (when he was of sufficient size) and began hemodialysis in preparation for kidney transplantation. To establish the genetic basis of the CNS and identify variants in other genes that may have ramifications for kidney transplant evaluation and planning, the TruGenome Undiagnosed Disease Test by Illumina Clinical Services Laboratory was performed on the patient and both parents. Results of genetic testing revealed two variants in *NPHS1*—a previously described pathogenic frameshift variant (c.2606_2607dupCC p.Asn870ProfsTer36) [2] inherited from the patient's father and a missense variant of uncertain significance (c.2159 A > C p.His720Pro) inherited from the patient's mother. Both parents have no history of kidney disease.

**Figure 1:**
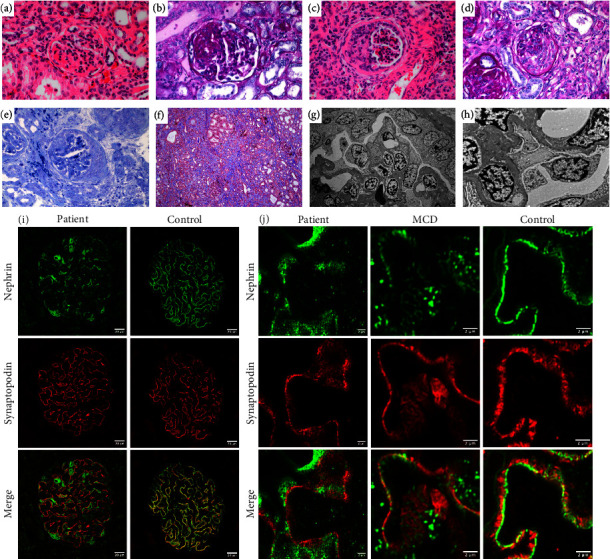
Histopathologic and immunohistochemical analysis of kidney sections from patient and controls. (a, b) Representative glomeruli showing FSGS: (a) H&E (400X) and (b) PAS (400X). (c–e) Representative glomeruli showing cellular crescent formation: (c) H&E (400X), (d) PAS (400X), and (e) Toluidine Blue (400X). (f) Representative section of the renal cortex illustrating severe interstitial fibrosis, trichrome (100X). (g, h) Representative images showing diffuse podocyte foot process effacement, electron microscopy (3000X and 8000X). (i) Immunofluorescence confocal microscopy comparing localization of nephrin and synaptopodin in the patient's kidney to a control kidney. (j) Airyscan immunofluorescence microscopy comparing the patient's kidney to MCD and control kidneys.

Histologic examination of paraffin-embedded tissue from the nephrectomy specimens via light, electron, and immunofluorescence microscopy revealed several notable findings. Light microscopy of H&E, PAS, Masson's trichrome, and toluidine blue stained sections showed focal segmental glomerulosclerosis (FSGS) and global glomerulosclerosis (5–10%), crescent formation, tubular microcystic changes, and severe interstitial fibrosis (Figures 1(a)–1(f)). Electron microscopy highlighted diffuse podocyte foot process effacement and normal glomerular basement membrane thickness without electron-dense deposits (Figures 1(g) and 1(h)). Localization of nephrin and synaptopodin in the patient's kidneys was compared to a control kidney via immunofluorescence confocal microscopy. Relative to the control, the nonsclerotic and noncrescentic glomeruli in the patient's sample showed nephrin aggregation in the podocyte cell body instead of a linear distribution along the glomerular basement membrane (Figure 1(i)). Nephrin colocalized with synaptopodin in a healthy control glomerulus, which was not observed in the patient's tissue (Figure 1(i)). To evaluate whether the changes in nephrin distribution were related to podocyte foot process effacement in general, a minimal change disease (MCD) sample was examined. Using Airyscan immunofluorescence microscopy, the nonsclerotic and noncrescentic glomeruli in the patient's sample showed strong nephrin staining in the podocyte cell body that was not present in the MCD or control kidney samples, indicating that the nephrin mislocalization is not simply a feature of diffuse podocyte foot process effacement as seen in MCD (Figure 1(j)). Both *NPHS1* variants cause alterations in the protein sequence prior to the transmembrane domain, suggesting that both likely cause trafficking defects, resulting in a failure of nephrin to incorporate into the cell membrane.

## 4. Discussion

CNS is an autosomal recessive disease usually attributable to defects in establishing and maintaining glomerular slit diaphragms. Most commonly, the disease is caused by homozygous variants in *NPHS1* [1, 2]. Initial presentation relates to the sequelae of significant proteinuria, which can include increased susceptibility to infections, thromboembolic complications, hypothyroidism due to loss of thyroxine-binding proteins, and general failure to thrive [1, 6]. In this case, the patient presented abruptly due to cerebral venous thrombosis. In addition, the patient has hypothyroidism and requires levothyroxine supplementation. Progression to kidney failure is variable but is often seen within the first three years of life [7]. Treatments are often supportive but can include early unilateral nephrectomy until the patient can undergo kidney transplantation [8]. Despite escalation in supportive care, the patient eventually required hemodialysis and a bilateral nephrectomy due to persistent massive proteinuria at two years of age. In those patients who receive a kidney transplant, nephrotic syndrome can recur post-transplant due to the development of anti-nephrin antibodies, which can require treatment with corticosteroids, cyclophosphamide, plasmapheresis, or anti-CD20 therapy [9]. Notably, genetic sequencing revealed compound heterozygous variants in *NPHS1*, a clinical scenario that has been more commonly described in late-onset FSGS but is now increasingly recognized in CNS [4, 5, 10]. The previously described pathogenic *NPHS1* frameshift variant inherited from the patient's father was identified in a patient who is also compound heterozygous for *NPHS1* variants [2].

This case of CNS had unique histopathologic findings in addition to those reported more frequently such as FSGS, tubular microcystic changes, and interstitial fibrosis. Glomerular crescent formation may be observed in patients with CNS as seen in this patient's case, but it is infrequent [11]. The absence of nephritic features argues for a different mechanism of crescent formation than those associated with rapidly progressive glomerulonephritis. Immunofluorescence microscopy for nephrin is negative in classic cases of CNS such as those patients who are homozygous for the Fin-major variant, which leads to a short, truncated protein. Staining depends on the antibody utilized. Both *NPHS1* variants found in this patient cause alterations in the protein sequence prior to the transmembrane domain and should be detectable by the nephrin antibody used in this report, which recognizes the extracellular domain of the protein. It is possible that one of the two variants has little to no expression due to protein stability. Nevertheless, compound heterozygosity with these two *NPHS1* variants causes mislocalization of nephrin. As illustrated by immunofluorescence microscopy, nephrin does not colocalize with synaptopodin in the patient's tissue, which is a marker of the podocyte foot process. Rather, nephrin appears to aggregate in the podocyte cell body. Nephrin missense variants can cause defects in intracellular transport [12]. Moreover, nephrin expression has been previously investigated in MCD with findings similar to those reported here [13]. The pattern of staining in this patient's case is distinct from that seen in MCD. Currently, there are limited data available to provide prognostic information regarding the various pathogenic nephrin variants, but this is an important avenue to explore in the future.

## 5. Conclusion

A combination of a known pathogenic frameshift variant in *NPHS1* and a previously undescribed missense variant in *NPHS1* resulted in continued expression but mislocalization of nephrin to the podocyte cell body instead of the slit diaphragm region. A MCD comparison sample did not show the same pattern, indicating that the mislocalization is not merely due to diffuse podocyte foot process effacement. Given that CNS caused by *NPHS1* variants is inherited in an autosomal recessive manner, the *NPHS1* missense variant (c.2159 A > C p.His720Pro) should be classified as pathogenic.

## Data Availability

The data are available on request to the corresponding author.
